# Supplementary material for: Predicting visual function by interpreting a neuronal wiring diagram
Source: Nature. 2024 Oct 2;634(8032):113–23. doi: 10.1038/s41586-024-07953-5 (PMC11446822; doi:10.1038/s41586-024-07953-5)
Supplement: Supplementary file 6 — CRF and ERF predictions for individual TmY4 and TmY9 cells. Analogous to Supplementary Data 3, but for TmY target types. Shown are the top four monosynaptic pathways, the strongest pathway passing through each of the top ten intermediary types (ranking from Extended Data Fig. 7), and the trisynaptic pathway Tm1–TmY–Dm3–TmY (see the section entitled Prediction of spatial normalization). [file 41586_2024_7953_MOESM6_ESM.zip › DataS4/TmY4/720575940633860494.pdf]

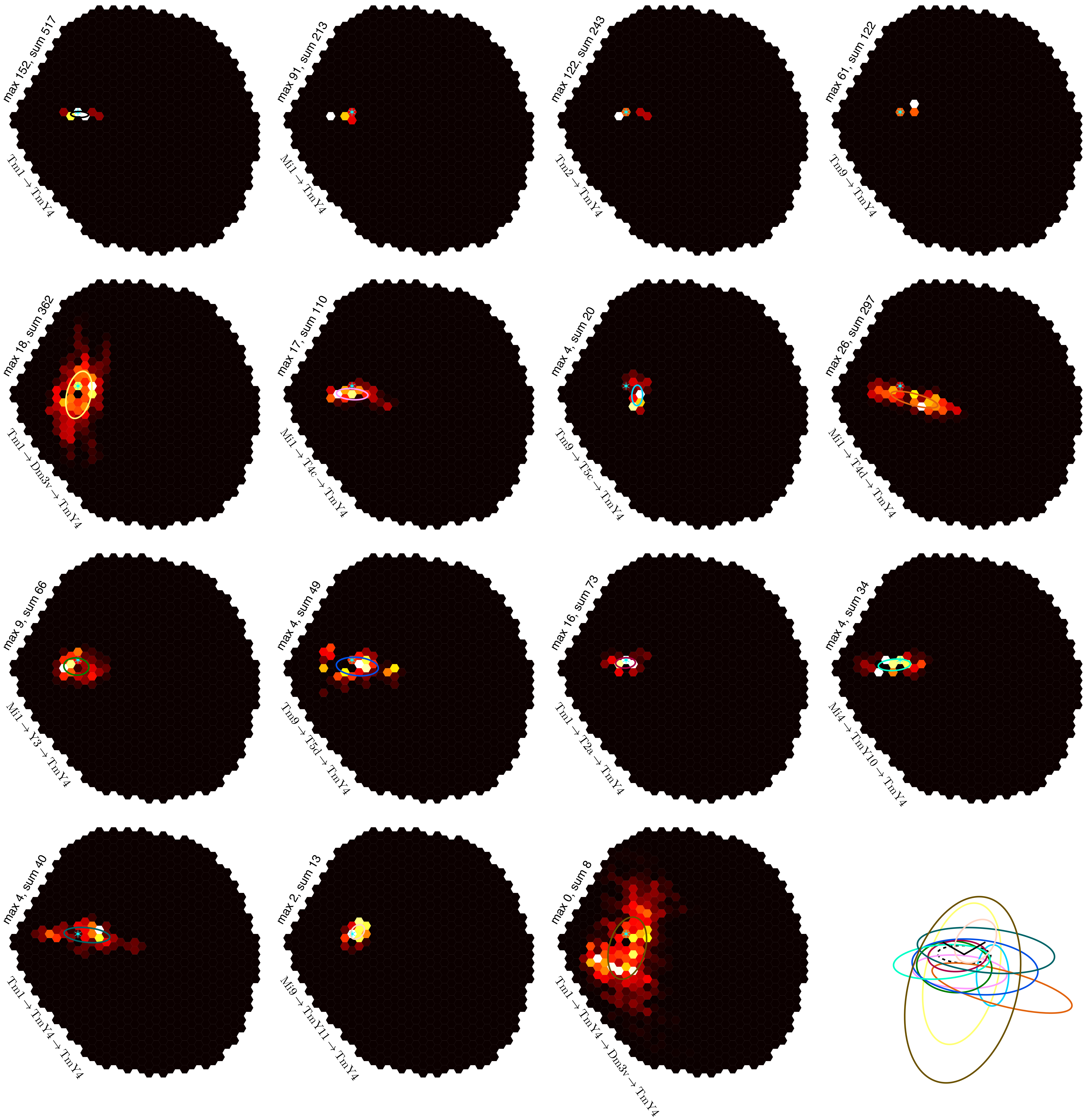
max 152, sum 517
Tm1 → TmY4
max 91, sum 213
Mi1 → TmY4
max 122, sum 243
Tm2 → TmY4
max 61, sum 122
Tm9 → TmY4
max 18, sum 362
Tm1 → Dm3v → TmY4
max 17, sum 110
Mi1 → T4c → TmY4
max 4, sum 20
Tm9 → T5c → TmY4
max 26, sum 297
Mi1 → T4d → TmY4
max 9, sum 66
Mi1 → Y3 → TmY4
max 4, sum 49
Tm9 → T5d → TmY4
max 16, sum 73
Tm1 → T2a → TmY4
max 4, sum 34
Mi4 → TmY10 → TmY4
max 4, sum 40
Tm1 → TmY4 → TmY4
max 2, sum 13
Mi9 → TmY11 → TmY4
max 0, sum 8
Tm1 → TmY4 → Dm3v → TmY4
